# Dying, death and bereavement: developing a national survey of bereaved relatives

**DOI:** 10.1186/s12904-023-01135-2

**Published:** 2023-02-23

**Authors:** Diarmuid Ó Coimín, Daniela Rohde, Conor Foley, Tracy O’Carroll, Róisín Murphy

**Affiliations:** 1 Hospice Friendly Hospitals Programme, Quality and Patient Safety Directorate, Mater Misericordiae University Hospital, Eccles Street, Dublin 7, Ireland; 2 National Care Experience Programme, Health Information and Quality Authority, George’s Court, George’s Lane, Smithfield, Dublin 7, D07 E98Y Ireland

**Keywords:** End-of-life care, Palliative care, Quality of care, Bereaved relatives, Bereavement, Dying, Survey of bereaved people, Mortality feedback survey

## Abstract

**Background:**

Assessing and measuring the experience and quality of care provided is central to the improvement of care delivery of all healthcare systems. This paper reports on the development of a survey instrument to capture the experiences of care at end of life from the perspective of bereaved relatives in the Republic of Ireland.

**Methods:**

A multi-method, multi-stakeholder, sequential approach was adopted for this study. Items for inclusion in the survey instrument bank were identified through (1) a feasibility study and scoping literature review, (2) expert panel programme board review, (3) focus groups and (4) gap analysis. The following steps were undertaken to prioritise the items for inclusion in the final survey instrument: (1) a Delphi study (2) technical expert panel review (3) cognitive interviews with bereaved relatives and an (4) expert panel programme board review.

**Results:**

Following an iterative process with key stakeholders, a survey instrument was developed with sections focusing on the provision of care at home, in the last nursing home / residential care facility, hospice and hospital, as well as care experience in the last 2 days of life, the relative’s experiences of care and support, the circumstances of care surrounding death and demographic information. In total, a bank of 123 questions were prioritised to be included in the National End of Life Survey instrument.

**Conclusion:**

The survey will provide a standardised national approach to capturing the experience of care of those who have died, from the perspective of bereaved relatives in the Republic of Ireland. This will allow health service providers, policy makers and regulators to gather important insights into the experiences of care at end of life and will help fulfil the requirement of healthcare services to ensure they are providing high-quality care.

**Supplementary Information:**

The online version contains supplementary material available at 10.1186/s12904-023-01135-2.

## Background

### Context

Palliative care is an approach to improving the quality of life of people facing a life-threatening illness and of their families. Palliative care focuses on four domains of care: the treatment of pain; symptoms experienced other than pain, as well as addressing and supporting psychosocial and spiritual needs [1]. Assessing and measuring the experience and quality of care provided is a key component of healthcare systems. However, there is no standardised national approach in Ireland to capturing the experience of care of those who have died and that of their relatives [2, 3]. Surveying people who are likely to die or who are actively dying poses significant challenges for numerous reasons, including the difficulty associated with predicting death [4, 5] as well as the sensitivity and ethical issues related to burdening a person dying in participating in a survey [6]. Other matters that have precluded people’s ability to participate centre on the frailty of the individual or inability due to being in a semi-conscious state and deterioration in condition [7–9]. Instead, surveying bereaved relatives has been recommended to provide important insights and understanding of the experiences and quality of care delivered at end of life [10–12]. Given this, studies concerned with the experience of end-of-life care have focussed on ascertaining the views of bereaved relatives on the care delivered to the person that died and those closest to them [13–17]. Internationally, surveying bereaved relatives is a recognised way of capturing care experiences to inform quality improvements in healthcare in England [14, 15, 18], the United States and Japan [19, 20].

### Policy context in Ireland

In 2020, 31,765 deaths were registered in Ireland, with 26,440 deaths of persons aged 65 years and over, accounting for 83% of all deaths registered that year [21]. More than two thirds of all people being cared for at end of life will die in a healthcare facility, with 43% dying in hospitals in Ireland [22]. National guidance, reports and policy documents recognise the importance of the provision of good care at end of life in Ireland [23–29]. Surveying bereaved relatives is recommended as a means of evaluating the experience of care delivered as outlined in the Irish Health Service Executive, National Clinical Care Programme for Palliative Care. This Programme makes a number of recommendations including seeking feedback from service users and relatives to inform improvement plans [30]. The Programme’s Adult Model of Care report recommends surveying bereaved relatives to measure the quality of life and death with a view to having ‘an improved patient experience and better quality of life and death’ and also to ascertain if people are ‘cared for in a place of care that is acceptable to them and their families’ [23].

The Irish Government commissioned a report in 2016 to examine how state services dealt with issues of dying, death and bereavement, which proposed that the State should conduct a national dialogue of end of life issues and noted the importance of engaging in a ‘listening exercise to learn about people’s direct experience of end of life’ [31]. The Programme for Government stressed the importance of service user engagement and committed under the heading ‘More Compassionate Care’ to ensuring ‘patients’ voices are heard’. The Programme commits to developing end of life services, noting ‘the care and dignity of a dying person and their family must be our focus’ [32]. Sláintecare, the cross-party strategy for health reform in Ireland, advocates the use of standardised national experience surveys to inform improvements and shape policy [33].

The National Care Experience Programme (NCEP) is a partnership between a health services regulator (Health and Information Quality Authority, HIQA), the Irish national healthcare service provider (Health Service Executive, HSE), and the Department of Health, the government’s principal advisor on health policy, governance, and performance oversight of the health sector. The NCEP undertakes surveys to systematically gather data on the experiences of health and social care service users in Ireland.

This paper describes the process used to develop a survey instrument for the first National End of Life Survey. The National End of Life Survey will aim to establish the quality of healthcare delivered by health and social care services to people approaching the end of their life and their relatives in Ireland. The findings of the National End of Life Survey will inform quality improvement initiatives, national standards, and monitoring programmes, as well as national policy and legislation.

## Methods

### Survey instrument development

A structured approach to the development of the National End of Life survey instrument was undertaken mirroring established methods utilised in the development of the National Inpatient Experience Survey and National Maternity Experience Survey [34–36]. Central to this work was the involvement and exploration of the experiences of key stakeholders involved in care at end of life in Ireland.

The content and design of the survey instrument were informed by eight sequential steps. The initial steps identified items for inclusion in the survey bank and explored the policy context and different methodologies used to date through a feasibility study, scoping literature review, expert panel review, focus groups and a gap analysis. The next steps centred on prioritising the items for inclusion in the final survey instrument through a Delphi study and cognitive interviews with bereaved relatives and a review by a technical expert panel, NCEP team and programme board members.

The development of the National End of Life Survey was governed by a Programme Board whose membership is made up of representatives of the NCEP partners, along with representatives of bereaved people, family carers and subject matter experts in palliative and bereavement care. The Programme Board recommended the development of a survey instrument specifically for use in the Republic of Ireland, to ensure that the policy and priorities of palliative care and end of life and bereavement care in the Republic of Ireland are reflected in the survey instrument. The development of the survey was led by an NCEP team whose membership included but is not limited to the authors of this paper.

### Participant recruitment

Participants recruited for focus groups and Delphi study were sampled purposively from a range of professional backgrounds with a broad array of roles and sectors represented, including bereaved relatives, specialist palliative care staff in direct care and management roles, general practitioners, medical consultants and clinical medical directors, nursing staff based in the community, nursing homes, hospices and acute hospitals in direct care and management roles, home care support staff, health and social care professionals, academic staff with a knowledge of palliative and end of life care, policy makers, funders and regulatory staff. Organisations supporting ethnic minority communities’ participation in health planning and delivery were also asked to nominate representatives, thereby ensuring participants recruited were from a range of diverse cultural and demographic backgrounds. An overview of the key stakeholders who participated, their backgrounds and roles within their organisations in the different stages of the survey’s development is outlined in supplementary information table 1. Participants for the focus groups, Delphi study and the individual cognitive interviews received an invitation by email, which included a comprehensive Participant Information Leaflet that provided details on the context and background of the study, aims of the work, what taking part in the study involved. In view of the potentially emotive nature of the subject, all participants received information on bereavement supports and participants were aware of the voluntary nature of participation and the right to withdraw from the study at any point. In the case of the Delphi study, the invitation email contained an electronic link to the information and consent page of the Delphi study. Participants were asked to tick a box to indicate their consent to participate, without which they were unable to proceed to the Delphi study questions. The inclusion of a wide range of stakeholders and particularly bereaved relatives ensured that the instrument reflected the experiences and aspects of care that were identified as being most important by all groups.

### Identification of possible items for inclusion in the survey instrument

#### Feasibility study

The first step involved in the development process centred on an exploration of Ireland’s national policies, guidance, standards, and regulatory standards relating to the provision of palliative and end-of-life care and which recommended utilising experience of care or surveys of bereaved relatives for the purposes of quality improvement. This step explored the feasibility of different mechanisms for gathering information about care at end of life from bereaved relatives in the Republic of Ireland. Comprehensive searches were undertaken to identify surveys of bereaved relatives conducted in Ireland, including the aims of the surveys, methods adopted, and inclusion and exclusion criteria. The feasibility study also explored and consulted with a wide range of stakeholders to examine the possibility of accessing a national data set with details of those who have died and contact details of their relatives. The methods used to identify the target population for the survey as well as the most appropriate methodological approach to inform the survey design and scoping literature review were also examined.

#### International literature review

A scoping review of international literature on bereaved relatives’ experience surveys was undertaken to inform the development of the survey instrument. This review consisted of a comprehensive electronic search of the PubMed Database, internet sources and a limited follow-up of cited references. Literature including technical reports and national policy documentation were also reviewed. This review found that surveying bereaved people is undertaken by many national public health agencies, healthcare providers and academic institutions internationally. Most surveys use data from bereaved relatives’ experiences of care for the purpose of evaluation of care at end of life. Surveys from several countries were reviewed. Four countries (the USA, England, New Zealand and Japan) were selected for this review based on the criteria that the country conduct surveys of bereaved relatives that are underpinned by legislation or health policy and/or undertake such surveys to evaluate the provision palliative care or care at end of life on a national basis. Findings for this scoping review were compiled on each country, from literature including technical reports, national policy documentation and peer reviewed journal articles. Detailed discussions with key personnel with responsibility for management of bereaved relatives experience surveys within each country, where available also took place.

The review examined the model and methodology associated with surveys of bereaved relatives developed and utilised internationally to ascertain the experience of care at end of life. This review focused on the context, key themes assessed, and methods employed to provide a synthesised knowledge of existing approaches and instruments for assessing the quality of end of life care from the perspective of bereaved relatives. The review examined the governance structures, model, and methodology and reporting processes.

#### Expert panel programme board review

An expert panel was convened, which consisted of members of the NCEP partners, including experts and representatives of palliative and bereavement care, bereaved relatives, policy makers, funders, and regulation staff. This panel met on two occasions to discuss and consider the findings of the feasibility study and literature review to inform the next steps of the survey instrument’s development and decide on the survey population and proposed methods of data collection for the National End of Life Survey in advance of the focus groups with key stakeholders.

#### Focus groups

Eleven focus group interviews were undertaken with stakeholder groups to explore the aspects of care considered to be most important for inclusion within the National End of Life Survey instrument. Focus group participants were sampled purposively from a range of professional backgrounds with a broad array of roles and sectors represented, including bereaved relatives. Due to restrictions on meeting in person as a result of the COVID-19 pandemic in March 2021, participants were informed from the outset that the focus groups would be held online by video conference call. Participants were allocated to homogenous groups to enhance group interaction, dynamics and confidence in sharing knowledge and understanding [37].

Focus groups were undertaken online through video conferencing call and lasted between 1 hour and 30 minutes to 1 hour and 45 minutes and were facilitated by the NCEP study team. The focus group discussions centred on the following two questions:

- What aspects of care during the last 3 months of life are the most important for inclusion in a survey of bereaved relatives?
- What aspects of care during the last 2 days of life are the most important for inclusion in a survey of bereaved relatives?

The facilitator presented a list of themes and domains of care that were identified through the scoping review of surveys of bereaved relatives. Based on this list, participants were asked to identify the most important theme to be included in the proposed survey instrument. Prior to the conclusion of each focus group, an open-ended question was asked to ascertain if participants had anything further to add or anything else that should be included in the survey instrument. These discussions clarified what matters most in the provision of end-of-life care in the Republic of Ireland from the perspective of key actors in that process.

Comments were transcribed verbatim for analysis. Transcripts were anonymised and checked with NCEP team members present at the focus groups for accuracy. Qualitative data were managed in Microsoft Excel and analysed using thematic analysis [38]. Initially 28 codes relating to various domains and themes of care at end of life were identified by the lead author. Similar codes were then merged, resulting in 7 overarching themes. This data was reviewed and confirmed by the authors as being the domains and themes of care that focus group participants identified as being most important for inclusion in the survey instrument.

#### Gap analysis

The next stage of the survey instrument’s development centred on mapping the suite of international questions identified in the literature review against Ireland’s national policies, guidance and standards relating to the provision of palliative and end-of-life care [23, 30, 39], national regulatory standards [40, 41], and the findings from the focus groups. The purpose of the gap analysis was to identify areas that were not captured within the international suite of questions or themes from focus groups but identified as important within the context of palliative care policy, standards, or service provision in the Republic of Ireland. In cases where themes were not sufficiently addressed by the questions from international surveys, a gap was identified. To address the gap, new survey questions were developed.

#### Prioritising items for inclusion in the final survey instrument

The prioritisation of items for inclusion in the final survey instrument centred on four sequential stages: 1. a two round Delphi study, with consensus on the final survey instrument bank of questions achieved through 2. a technical expert review, 3. cognitive interviews with bereaved relatives; and 4. a review by the NCEP study team and expert programme board members.

#### Delphi study

A two round Delphi study was undertaken with key stakeholders to prioritise the questions for inclusion in the final survey questionnaire. This technique is commonly used to gain consensus among an expert panel through an iterative process involving a number of rounds, with information and results synthesised and fed back to panel members between rounds [42]. Delphi studies are particularly useful for problems that benefit from subjective judgements on a collective basis, when experts are not directly in contact with each other, where the number of experts is too large to interact face-to-face or where ethical or social dilemmas dominate economic or technical ones [43, 44]. The purpose of the Delphi study was to ensure that the most important areas of care were included, while maintaining a reasonable survey length to avoid overburdening respondents. The Delphi study was undertaken using an online structured questionnaire, administered using web-survey software. Participants were also afforded the opportunity to provide qualitative feedback on the content and wording of items [45]. Potential participants were recruited by the lead author and sampled purposively from all key stakeholder groups involved in the focus groups.

For round one, participants were asked to rate each potential survey question on its importance for inclusion in the final National End of Life Survey instrument on a 5-point Likert scale (from ‘strongly disagree’ to ‘strongly agree’ that the item should be included). The first round contained 145 items, of which 21 routing or demographic questions were deemed essential and included for the participants’ information only. Following round one, items that failed to reach a threshold of 77% or above of ‘agree’ or ‘strongly agree’ responses were removed. For the second round, participants were asked to rate questions based on whether or not each question should be included in the final questionnaire on a 10-point rating scale (from ‘definitely no’ to ‘definitely yes’). Questions that achieved a mean rating of < 8 were removed. Given the high degree of agreement among stakeholders on the importance of each remaining survey item (all remaining questions had a median score of 9 or 10), further rounds were deemed unnecessary.

#### Technical expert review

The next stage in the prioritisation and selection of items to be included in the survey instrument centered on a technical expert review of the questions. Picker Institute Europe, a leading healthcare agency with technical knowledge and expertise in developing and researching patient and staff experience of care, were commissioned by the study team to review the survey instrument prior to cognitive testing with bereaved relatives.

#### Cognitive interviews

The aim of the cognitive interviews with bereaved relatives was to ensure sequence, flow and clarity of questions and responses. Participants were recruited by the lead author and sampled purposively from bereaved relatives, some of whom had participated in other elements of the survey instrument development such as the focus groups or Delphi study and had consented to be contacted to participate in the cognitive testing. Others were recruited through an open call to voluntary agencies representing bereaved relatives.

Cognitive interviews were undertaken by videoconference call and lasted between sixty and ninety minutes. Potential participants received an invitation by email, including a detailed Participant Information Leaflet and bereavement support information. All participants gave written informed consent prior to interviewing. Participants were asked to read through the survey questions and response options and comment on their interpretation and understanding of questions including whether the list of response options was exhaustive. Participants were also asked their views on the layout, length and its acceptability for completion, and overall views of the survey instrument. Participants were also asked to identify what if any improvements could be made to the survey instrument. The lead author transcribed comments verbatim for analysis. Transcripts were anonymised and checked with a co-author present at the cognitive interview for accuracy. Responses were analysed using thematic analysis [38]. Qualitative data were managed in Microsoft Excel and coded by one author. Four codes were identified by the NCEP study team.

## Results

**Fig. 1:**
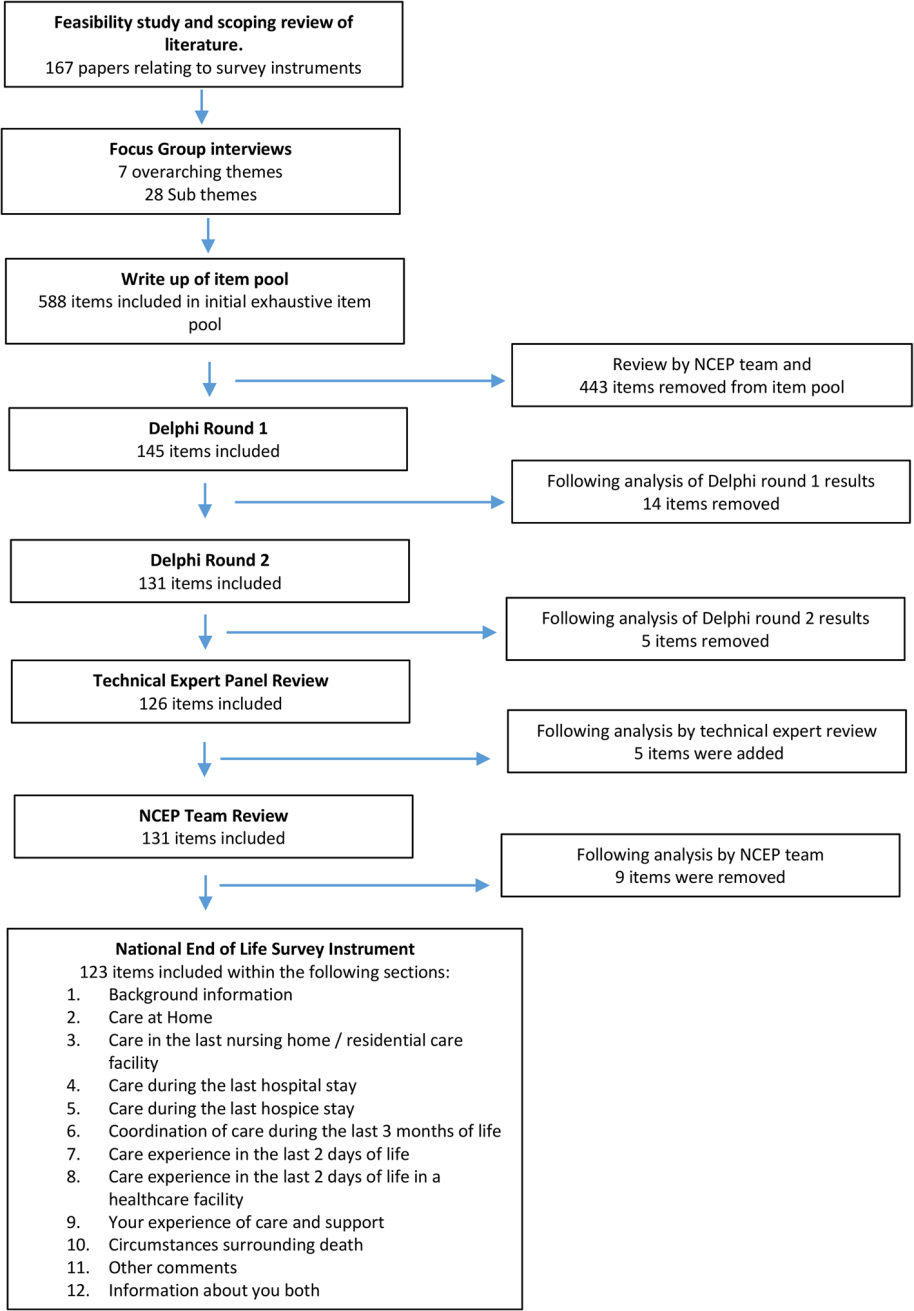
Overview of the survey instrument development process

An overview of the survey development process and results of this work are presented in Fig. 1.

### Identification of possible items for inclusion in the survey instrument

#### Feasibility study

A number of hospitals and hospices have undertaken surveys of bereaved relatives through collaboration with voluntary agencies or academic partners for research or quality improvement purposes [16, 46–48]. Previous surveys of bereaved relatives in Ireland were developed to evaluate care at end of life for adults who died in specific settings such as hospitals [16, 46], while some instruments were developed for adult populations outside of the Republic of Ireland, for example in hospices with different health care policy, regulations and national standards [47, 48]. The Survey of Bereaved Relatives: VOICES MaJam was the largest survey of bereaved relatives in Ireland, undertaken in two adult acute hospitals for the purposes of quality improvement. The VOICES MaJam report specifically recommended that surveys should be undertaken on a national basis to aid understanding of the experiences of care provision at end of life and support benchmarking of end-of-life quality improvement initiatives in Ireland. The report also suggested that the structures and expertise to undertake this work already existed within the Irish healthcare system, identifying the National Care Experience Programme as one source that could lead on this work [2, 3, 16]. The Office of the Ombudsman, whose role is to examine complaints from members of the public, recommended health service providers to proactively undertake surveys of bereaved relatives to provide insight into service provision and care at end of life for the purposes of quality improvement in Irish healthcare services [24, 49]. Government and health policy, and research has therefore endorsed engaging bereaved relatives to improve the quality of care at end of life provided by health and social care staff [2, 16, 23–25, 28].

#### Literature review

The review found that surveys of bereaved relatives are undertaken by many national public health agencies, healthcare providers and academic institutions internationally [50]. Most surveys use data from bereaved relatives’ experiences of care for the purpose of evaluation of care delivered at end of life to adults [6, 10, 13, 14, 50–52]. Some surveys of bereaved relatives are solely focussed on the provision of care of the family and adult in the last days of life [53, 54]. Other surveys take a broader outlook, focussing not only on the provision of good palliative care in the days immediately preceding the time of death, but also care experiences in the weeks and months before the person dies, including care of their relatives at this time [19, 55–58].

The quality of care and the time period assessed varied across the survey instruments reviewed, with some focused on the quality of care delivered during the last admission within a particular healthcare setting such as a hospice or hospital, while other instruments focussed on the care provided by a particular service such as palliative care [19, 20, 55–61]. Some surveys took a wider population-based approach and included all bereaved people of those who died within a particular time frame, for example the last 3 months of life, using death registration or a national health data set to access the sample population [15, 50, 51, 62–64]. The mode of administration varied across surveys reviewed, with the majority utilising a questionnaire and postal mode of administration including reminders to non-responders [15, 20, 59–61, 64, 65]. An exception to this is the National Audit of Care at End of Life in England, where the method of response is online only with no reminders being sent [13]. Some surveys also offered the opportunity to respond by telephone [19, 66]. An overview of international surveys of bereaved relatives, their objectives, population surveyed, reporting outputs of each survey and their operational status is outlined in supplementary information Table 2. Further details on the methods and results of this review are published elsewhere [50]. The review informed the identification of the target population for the survey, as well as the most appropriate methodological approach.

#### Expert panel Programme board review

Prior to undertaking further work on the survey instrument development, the expert panel Programme Board was convened and met on two occasions to review the next steps and recommended based on the evidence of the work undertaken that the survey instrument should:

- be a population based survey if access to the national death registration data set is made available to undertake this work, similar to surveys undertaken in England [15, 64], Japan [20, 59–61] and New Zealand [50, 62, 65–67].
- seek to evaluate the experience of care delivered in the last 3 months of life as this is a defined period which is viewed as important for people approaching end of life and also care in the last days of life [6, 15, 50, 62, 64–69]
- seek to evaluate the experience of care in all settings of care at end of life; that is home, hospital, nursing home / residential care facilities and inpatient hospice [15, 50, 62, 64–69]
- seek to review the experiences of care associated with the deaths of adults only, therefore excluding the deaths of children. This was based on the international evidence that all surveys reviewed excluded children [15, 19, 20, 50, 59–61, 64, 65]. Exclusion of children also centred on the rationale that adult health service provision, health policy and bereavement supports services differ significantly to those of children in the Republic of Ireland [70, 71].
- exclude people who died suddenly and unexpectedly, due to the late registration of people who died suddenly or traumatically by suicide or homicide due to coronial investigations, as well as the fact that questions focussed on the quality of care delivered in the time leading up to the death would be irrelevant to this population [6, 15, 21, 50, 64, 65, 68, 69].

Based on the scoping review and feasibility study, the expert panel proposed the following with regard to mode of administration based on the international [50] and national [2, 46] evidence that this is acceptable to bereaved relatives noting the sensitive nature of such a survey:

- adopt a questionnaire and postal mode of administration to bereaved relatives
- no active solicitation or contact with bereaved relatives that registered the death would take place until 3 months after the death
- the inclusion of bereavement support information in all correspondence related to the survey’s administration
- to increase response rates, include two reminders to non-responders with a survey questionnaire also included with the second reminder
- include an option for participants to complete an online version of the questionnaire.

#### Focus groups

Eleven Focus group interviews were undertaken with a purposive sample of key stakeholders to explore the aspects of care considered to be most important for inclusion within the National End of Life Survey instrument. Sixty eight people took part from a broad range of roles, professional backgrounds and sectors as outlined in outlined in supplementary information table 1. Numerous themes were identified as being important for inclusion in each of the different settings of care (home, acute hospital, nursing home / residential care facility and inpatient hospice unit). Table 1 outlines the overarching themes and subthemes, the frequency with which subthemes were mentioned by focus group participants relating to what matters most for inclusion in the survey instrument according to the key stakeholders who participated in the focus groups.

**Table 1:** Themes identified by focus group members

Major theme		Sub themes	N
1. Physical care and comfort	1	Symptom management	**10** out of 11 focus groups
	2	Pain management	**11** out of 11 focus groups
	3	Personal care and nutrition	**8** out of 11 focus groups
	4	Unnecessary medical interventions	**3** out of 11 focus groups
	5	Community services and practical resources	**7** out of 11 focus groups
	6	Urgent care out of hours	**2** out of 11 focus groups
2. Psychosocial care	7	Emotional care and support	**11** out of 11 focus groups
	8	Psychosocial care and support	**10** out of 11 focus groups
	9	Financial or legal matters	**9** out of 11 focus groups
3. Spiritual and Cultural Care	10	Spiritual and religious care and support	**11** out of 11 focus groups
	11	Cultural care	**2** out of 11 focus groups
4. Person centred care and support	12	Holistic care and support	**11** out of 11 focus groups
	13	Person treated with compassion and kindness, dignity and respect,	**10** out of 11 focus groups
	14	Confidence in staff skills and knowledge	**4** out of 11 focus groups
	15	Continuity of care between care settings	**9** out of 11 focus groups
	16	Route of admission	**7** out of 11 focus groups
	17	Coordination of care	**2** out of 11 focus groups
5. Communication	18	Importance of clear and comprehensive communication of information	**11** out of 11 focus groups
	19	Communication about impending death	**11** out of 11 focus groups
	20	Access to palliative care	**2** out of 11 focus groups
	21	Care after death	**6** out of 11 focus groups
	22	Advance care planning	**9** out of 11 focus groups
	23	Preferred place of care /preferred place of death	**9** out of 11 focus groups
6. Physical Environment	24	Importance of care in a single room compared to multi-occupancy room	**9** out of 11 focus groups
	25	Facilities for relatives - family rooms	**8** out of 11 focus groups
7. Support for relatives	26	Support and services for relatives including bereavement support	**10** out of 11 focus groups
	27	Family presence - being able to visit at any time	**10** out of 11 focus groups
	28	Family provision of care and support	**4** out of 11 focus groups

#### Gap analysis

In total, 12 survey instruments were reviewed within the gap analysis with a total of 588 survey items:

- End of Life Care Provision by South Island Health Care Services survey and Auckland District Health Board, Survey of Bereaved People (VOICES 2017), New Zealand, (2018)
- National Audit of Care at the End of Life (NACEL), England, (2021)
- National Survey of Bereaved People (VOICES), England, (2015)
- Care of the Dying Evaluation (CODE) survey, England, (2013)
- FAMCARE survey, Association for Palliative Medicine of Great Britain and Ireland, (2019)
- Consumer Assessment of Healthcare Providers and Systems (CAHPS) Hospice survey, Centers for Medicare & Medicaid Services, United States Department of Health and Human Services, USA, (2020)
- Bereaved Family Survey (BFS) United States Department of Veterans Affairs, USA, (2019)
- Japan Hospice and Palliative Care Evaluation (J-HOPE) surveys consisting of the Care Evaluation Scale (CES) and the Good Death Inventory (GDI), Japan, (2020)
- Survey for Relatives / Friends on End of Life Care, Ireland, (2014)
- Survey of Bereaved Relatives: VOICES MaJam, Ireland, (2017)

Using a tabular format, analysis was undertaken through the identification of all aspects of care that must be included within the Irish survey instrument as per five documents related to the provision of palliative and end-of-life care in Ireland and HIQA regulatory standards (as above) and the themes from the focus groups (Table 1).

A number of specific areas raised by focus group participants were partially reflected in the international survey instruments reviewed. In cases where themes were not sufficiently addressed by the questions from the survey instruments reviewed, a gap was identified. To address these gaps, new survey questions were developed, including questions on the provision of care in a single room or multi-occupancy room at end of life, facilities for families such as designated family rooms to meet healthcare staff in privacy, open visitation of relatives in healthcare settings, and questions related to the support provided to relatives in the last days of life and at the time of death.

#### Questionnaire compilation

The first step in compiling the questionnaire centred on collating the themes identified through the focus group interviews, international review and identified policy, standards, and gap analysis. In line with the recommendations of the expert panel, the questionnaire covered the main places of care where a person with a life limiting illness may have been cared for during the last 3 months of life, with domains of care replicated across each place of care. The survey instrument uses skip logic as not all items are relevant to all bereaved relative respondents, thereby minimising the burden on respondents.

The questionnaire sections were structured to follow a logical sequence:

1. Background information
2. Care at Home
3. Care in the last nursing home / residential care facility
4. Care during the last hospital stay
5. Care during the last hospice stay
6. Coordination of care during the last 3 months of life
7. Care experience in the last 2 days of life
8. Care experience in the last 2 days of life in a healthcare facility
9. Your experience of care and support
10. Circumstances surrounding death
11. Other comments
12. Information about you both

Three free text open-ended questions were included in the section titled ‘other comments’ based on the experience of previous research and other survey instruments, which have reported that the inclusion of open-ended questions facilitated and provided rich insights into the quality of care at end of life from respondents for the purposes of quality improvement [2, 3, 72].

Based on the international review of surveys of bereaved relatives and to reflect the sensitivity of the questionnaire’s content, the sample questionnaire used the third person pronoun (e.g., he / she) to avoid confusion and differentiate between the person that died and the person responding. This has been adopted in several studies and mirrors the work of the VOICES survey, the Bereaved Family Survey and CaregiverVoice survey and is deemed to be sensitive to bereaved relatives [6, 15, 50, 55–58, 64, 65, 68, 69, 73].

### Prioritisation of items for inclusion in the survey instrument

#### Delphi study

A two round Delphi study was undertaken with key stakeholders to prioritise the questions for inclusion in the final survey instrument. One hundred sixty three people participated in round one, with 82% of people (*n* = 134) indicating that they had experienced the death of a close family member and 37% (*n* = 61) stating they worked in a specialist palliative care service. In round two, 141 people took part, with 85% of people (*n* = 121) indicating that they had experienced the death of a close family member and 34% (n = 61) stating they worked in a specialist palliative care service. Participants came from a broad range of roles, professional backgrounds and sectors as outlined in outlined in supplementary information table 1. Fourteen items were removed following round one, including two questions related to the provision of night nurse services at home and three questions relating to transitions of care and advance care planning. A further five items were removed following Delphi study round 2, including questions relating to the provision of home help support at home (e.g., ‘During the last three months of her life, while she was cared for at home, did she get enough help and support from a paid carer (sometimes called home help or care assistants’).

Qualitative feedback was provided by ten people following round one and six people on completion of Delphi study round two. Participants strongly endorsed the content and clarity of questions. Some participants suggested minor rewording of questions to enhance participants’ understanding. For example, use of the term ‘member of public health nursing team’, which captures the care provided by public health nurses and community based registered nurses instead of the term ‘public health nurse’. The authors (DÓC and CF) discussed the qualitative feedback and questions were reworded in advance of the expert review and cognitive interviews with bereaved relatives.

#### Technical expert review

The Picker Institute Europe team reviewed the structure, content and flow of the questionnaire and made a number of suggestions to enhance the survey instrument questions. Two authors (DÓC and CF) discussed the feedback from the Picker team technical expert review, and questions and responses in the instrument were amended or reworded to reflect this feedback. This included simplifying questions that originally had two interlinking questions into separate individual questions and responses to aid understanding and completion by participants. Other suggestions centered on the alignment of responses with other National Care Experience Programme surveys. A number of questions were reorganised in the survey instrument to separate items specifically focusing on the care experience of the respondent to that of the experiences of the person that died to avoid confusion.

#### Cognitive interviews

Two rounds of cognitive interviews of the questionnaire were conducted to assess the clarity and appropriateness of the content and length of the proposed National End of Life Survey instrument and associated documents (invitation letters and bereavement support information). Eight bereaved relatives took part in the interviews. The first round consisted of five participants with the second round being undertaken with three different participants who had not taken part in the first round. Participants represented the relatives of people who were cared for in the last months of life at home, in a nursing home, hospital and hospice or in multiple settings in the last months of life e.g., nursing home and hospital.

Participants in the cognitive interviews reviewed the content, length and comprehension favourably commenting on the clarity and sensitivity of questions and responses, the logical flow of the questions and ease of completion. Cognitive interview participants deemed the length and number of questions in the survey instrument as being acceptable for completion by bereaved people. Participants supported the sensitive use of pronouns which personalised the questionnaire and recommended the survey administration process including the receipt of a hardcopy questionnaire for those who may have difficulty with completing a survey online. Participants viewed the supporting documentation such as invitation letters and bereavement support information very positively commenting on the ease of understanding and conciseness of the bereavement information and its adequacy to meet a bereaved relatives needs and signposting to bereavement support services.

Interviewees also made a number of suggestions to improve the content of the survey instrument. For example, in round one, participants suggested that some of the demographic questions be moved to the beginning of the questionnaire as they felt that commencing the instrument with a question related to ‘duration of illness is too blunt and stark’. Participants also provided feedback on the invitation and reminder letters. A number of changes were made to the survey instrument following round one of cognitive testing. No significant modifications or additions were suggested following round two of testing, with a few minor changes suggested to the wording of responses. The questionnaire was amended and finalised, with a third round of cognitive interviews deemed unnecessary as no new issues were raised by the participants or suggestions made to modify the content of the survey instrument or the associated documentation. A copy of the National End of Life Survey final question bank is included in supplementary information Table 3.

#### Expert panel Programme board review

A final review was undertaken by the expert panel Programme Board. The panel members endorsed the key domains and themes of care experience proposed in the National End of Life Survey instrument.

## Discussion

Surveys of bereaved relatives are widely utilised by policymakers, funders, and healthcare service providers to ascertain the quality of care delivered at end of life for the purposes of quality improvement [7, 74]. The aim of this study was to develop a survey instrument to capture the experiences of care at end of life from the perspective of bereaved relatives with a view to providing insights into the care delivered in all settings of care for the purposes of quality improvement. Whilst several studies have developed or utilised survey instruments to report on the perceptions and experiences of care experience from bereaved relatives, they were focused on care within specific settings such as hospices [47] and hospitals [16, 46, 48]. This survey instrument was designed specifically to review the experiences of care on a population wide basis in all settings of care and is the first population based survey instrument designed to ascertain the experience of care at end of life in the Republic of Ireland, meeting national policy and strategy set out by the National healthcare services provider [23, 30] to undertake such surveys for quality improvement.

A structured sequential approach was adopted in the development of the survey instrument. The feasibility study and scoping literature review established the widespread use of such surveys internationally and the different contexts within which surveys of bereaved relatives were undertaken [50]. The literature revealed the significant variation of how bereaved relatives are identified, for example through the healthcare records of health care providers [13, 20, 58, 63] or accessing the details of bereaved relatives through a national death registration data set [6, 59, 64, 73]. It also identified the considerable variation regarding survey objectives, populations surveyed, the timing of survey instrument administration, reporting outputs of each survey and their operational status.

The feasibility study and review of articles informed the expert panel programme board decisions on the population to be surveyed including inclusion and exclusion criteria, the points in time of care to be evaluated, survey administration including methods of identifying bereaved relatives and methods to maximise response rates [50]. Decisions made by the expert panel programme board informed the next steps regarding the survey instrument’s development.

Seven overarching themes with 28 sub-themes were identified through the analysis of data from the focus groups, outlining the domains and aspects of care considered to be most important for inclusion within the National End of Life Survey instrument. The seven overarching themes centred on physical care and comfort, psychosocial care, spiritual and cultural care, person centred care and support, communication, physical environment and support for relatives. The focus group themes and subthemes reflect the themes and domains of care that have been identified in similar survey instruments [50]. While considerable heterogeneity in the content of such surveys was noted by Lendon et al. in their systematic review [7], the themes and domains of care consistently included in survey instruments reflect the core themes of good palliative care provision including pain and symptom management, the provision of emotional and psychosocial care and support and spiritual care and support in line with the WHO definition of palliative care [1, 7, 50]. The overarching themes identified in the focus groups also reflect the domains of care reported in Virdun et al.’s systematic review and Robinson et al.’s integrative review of issues that were important to patients and their families relating specifically to care in the hospital setting such as effective communication and expert care such as good symptom management [74, 75].

Participants in the focus groups and Delphi study also noted the importance of the physical care environment and specifically the inclusion of questions relating to the provision of care in a single room and facilities for families such as access to family rooms to enhance the communication and provision of care at end of life. The Institute of Medicine endorsed six dimensions of patient-centered care. This framework does not explicitly reference the importance of the physical environment, however, it is consistent with the dimension of good communication, emotional support and more specifically the importance placed on the involvement of family and friends [76]. This domain of care has been identified as an important factor in good care provision in previous research nationally [2, 46, 77]. Interestingly, environmental aspects of the healthcare settings were noted as important in the systematic reviews; however, were rarely assessed in the survey instruments reviewed and recommended for inclusion in future survey instruments [7, 74]. Building on the recommendation of the systematic reviews, findings from an integrative review and the data from the focus groups in our study, which strongly endorsed their inclusion, questions on the physical environment were deemed important for inclusion in the National End of Life Survey instrument [7, 74, 75].

Other domains of care rarely assessed in international survey instruments centre on the subdomains of care identified in the focus groups relating to advice and support regarding financial and legal matters and bereavement support for relatives. While there is a significant financial burden and impact for those diagnosed with a life limiting illness and their families [78], this domain of care is rarely captured by surveys of bereaved relatives, and has been recommended for inclusion [7, 74]. Based on the data from the focus groups and Delphi study, the National End of Life Survey instrument contains questions on both financial and bereavement support for relatives. The data from the focus groups therefore adds to and builds on existing research and our understanding of what is important to include in such survey instruments [7, 50, 74, 75].

The National End of Life Survey instrument focusses on the elements of care that were identified by multiple stakeholders as being important across the different settings of care and two different time points of care in the last 3 months of life and last days of life in an Irish context. This builds on existing work and the findings of a systematic review of surveys of bereaved relatives which reported on 51 survey instruments, noting that only the VOICES survey instrument examined the experiences of care across all settings of care and at two different time points [7]. This survey instrument’s development has generated important information and data from the perspective of multiple stakeholders including bereaved relatives’ views on what is important in the provision of care at end of life and what should be included in a survey instrument to capture the experiences of care at this time from bereaved relatives. International research has recommended enhancing palliative and end of life care through capturing information about the provision of care in homes, nursing homes and the increased provision of bereavement services [78]. Whilst this study is focused on the development of a survey instrument for use in the Republic of Ireland, it will support the capturing of such information and is likely to be of interest to those working in end of life and bereavement care internationally as the themes identified through the structured sequential process reflect the themes and domains of care that have been identified in similar survey instruments [6, 50, 64, 73]. In addition, it includes questions on theme of the physical environment in the provision of care at end of life which may inform the development of future surveys of bereaved relatives internationally. This work has generated an item bank of 123 questions that will be used as the basis of the National End of Life Survey. A copy of the National End of Life Survey final question bank is included in supplementary information Table 3, the data obtained through this surveys administration will be utilised for the purpose of quality improvement of healthcare services.

## Strengths and limitations of this work

Strengths of the study include a comprehensive and robust method to the development of a survey instrument containing the key elements identified by stakeholders as central to the provision of good care at end of life. Findings from this work through an iterative consultation process with key stakeholders build on and increase our knowledge of what is important in the provision of health and social care service to people at end of life and their relatives. The final item bank of questions broadly reflects items in similar surveys of bereaved relatives with a number of additions to reflect domains of care that were identified as being important for inclusion in the National End of Life Survey. This work therefore adds to and strengthens the international research in the context of surveying bereaved relatives for quality improvement purposes.

We acknowledge that the study is limited somewhat by representing the views of key stakeholders who were identified and subsequently chose to take part through convenience sampling. The limitations of convenience sampling are well versed in literature and acknowledge that by including the same participants we may not have captured as wide-ranging a view as we would have done with ‘new’ participants for each element of the development process. This is mitigated somewhat by the number and range of people who took part in the different elements. However, focus group participants and Delphi study respondents were represented from all settings of care such as community services, hospices, hospital, and nursing homes with a wide variety of staff and bereaved people from across the Republic of Ireland taking part. Representatives of people from ethnic minority groups were included in all stages of the survey instrument development, however, participants in focus groups, Delphi study and cognitive interviews who did not have a competent level of English were excluded from participation. This is acknowledged as a limitation to the development of the survey instrument. There is a growing body of literature reporting on the implementation of experience surveys with concerns that such information is not always used to improve care, a significant limitation of such surveys. Important lessons have been learned on bridging the gap between measurement and implementation of improvements which need to be considered to enhance care experience by healthcare services [79, 80]. Despite the above limitations, the data and resulting survey instrument represents the most important aspects of care to be included in a national survey of bereaved relatives in the Republic of Ireland.

## Conclusion

This paper describes the sequential process utilised to develop the survey instrument for the National End of Life Survey, as recommended by national policy and research. The development of a survey instrument specifically for use in the Republic of Ireland centred on ensuring that the policy and priorities of palliative care, end of life and bereavement care in Ireland are reflected in the survey instrument given the different context of care provision in other countries. The National End of Life Survey instrument reflects the key elements of care that are deemed as most important from multiple stakeholders including bereaved relatives. The survey will ensure a coordinated national approach to capturing the experience of care at end of life delivered by health and social care services for the purpose of quality improvement. The findings from the National End of Life Survey will inform quality improvement initiatives, national standards, and monitoring programmes, as well as national policy and legislation.

## Supplementary Information

**Additional file 1: Supplementary information Table 1.** Outlines an overview of participant’s backgrounds and roles within the different stages of the survey instrument development.

**Additional file 2: Supplementary information Table 2.** Outlines an overview of international surveys of bereaved relatives, their objectives, population surveyed, reporting outputs of each survey and their operational status.

**Additional file 3: Supplementary information Table 3.** Outlines the National End of Life Survey Final Question Bank.

## Data Availability

The datasets used and/or analysed during the current study are available from the corresponding authors on reasonable request.

## Abbreviations

HIQA: Health and Information Quality Authority
HSE: Health Service Executive
EoLC: End-of-Life Care
NCEP: National Care Experience Programme
