# Supplementary material for: Dying, death and bereavement: developing a national survey of bereaved relatives
Source: BMC Palliat Care. 2023 Feb 23;22:14. doi: 10.1186/s12904-023-01135-2 (PMC9947439; doi:10.1186/s12904-023-01135-2)
Supplement: Supplementary file 1 — Additional file 1: Supplementary information Table 1. Outlines an overview of participant’s backgrounds and roles within the different stages of the survey instrument development. [file 12904_2023_1135_MOESM1_ESM.pdf]

Supplementary information table 1:

Supplementary information table 1 outlines an overview of participant's backgrounds and roles within the different stages of the survey's development.

National End of Life Survey participants backgrounds and roles:	
**Administration / Management**	Chief Executive Officers of acute hospitals and hospice services, manager of community services, programme managers, manager of palliative care services, manager of advocacy organisation, home care support services manager, autopsy/mortuary manager
**Academia or Education**	Associate Professor, Co-Director, Professor, Researcher, training role with healthcare staff
**Bereaved people**	People who experienced the death of a family member or close friend
**Advocacy agency employee**	Development officers or those working to improve ethnic minorities' access to services and those working to improve palliative care and bereavement care in Ireland.
**Doctors**	Clinical director, clinical leads, consultants and registrars in medicine and palliative medicine, senior physician with responsibility for governance and policy and General Practioners (family doctor)
**Nurses**	Senior Nurse lead with responsibility for governance and policy, director of nursing, assistant directors, clinical nurse specialist, staff nurses, palliative care nurses, nursing staff working in adult acute hospitals, community hospitals, nursing homes and care at home
**Occupational Therapy, Physiotherapy, Pharmacy, Pastoral care**	Homecare and palliative care, adult acute hospital, palliative care and management
**Policy development**	Cancer, medicine, nursing, older persons, palliative care, patient safety and social care
**Regulation**	Inspectors
**Social Work, Psychology**	Bereavement, palliative care, medical social work
**Other**	Participants who classified as 'other' included those in roles such as bereavement volunteers or who didn't disclose background.
