# Supplementary material for: Dying, death and bereavement: developing a national survey of bereaved relatives
Source: BMC Palliat Care. 2023 Feb 23;22:14. doi: 10.1186/s12904-023-01135-2 (PMC9947439; doi:10.1186/s12904-023-01135-2)
Supplement: Supplementary file 2 — Additional file 2: Supplementary information Table 2. Outlines an overview of international surveys of bereaved relatives, their objectives, population surveyed, reporting outputs of each survey and their operational status. [file 12904_2023_1135_MOESM2_ESM.pdf]

Supplementary information table 2:

Supplementary information table 2 outlines an overview of international surveys of bereaved relatives, their objectives, population surveyed, reporting outputs of each survey and their operational status:

	Survey objectives and timeframe	Population	Survey Sample	Outputs	Current status
**England:** National Survey of Bereaved People (VOICES – Views of Informal Carers Evaluation of Services), 2011 to 2015 by the Office of National Statistics	▪ Inform national palliative and end-of-life care practice, policy and the provision of care services<br><br>Timeframe: reviewed care in **<u>last 3 months and last days of life</u>**	Adult population based survey across **all settings of care**	246,752 bereaved relatives received VOICES Survey in a 5 year period with 110,311 people responding – average of 45% response rate	<u>National Reports</u>: were published online outlining data associated with each section and question in the survey<br><u>Individual Reports:</u> No individual reports were developed as the VOICES survey data was not collected to report at individual healthcare provider level	The VOICES Survey has not been undertaken by ONS since 2015. The NHS evaluated the survey acknowledged data provided a useful a national picture for policy and service provision. NHS evaluation indicated that increased sample size was required to allow data to be collected to allow for individual reports for hospitals and other healthcare services to be developed for quality improvement purposes.
**England:** National Care of the Dying Audit for Hospitals (NCDAH) England 2013-2016	▪ assess compliance with national guidance on care at the end of life<br>▪ measure the experience of care at the end of life for dying	Adults who died in **acute hospitals**	NCDAH surveyed 2,313 bereaved relatives, with 858 respondents, a response rate of 37%	<u>National Reports and Individual Reports</u>: NCDAH: Report results in a national report with a separately published appendices outlining detail of individual hospital/trust level results.	National Audit of Care at the End of Life (NACEL), continue to undertake surveys of bereaved relatives of

National Audit of Care at the End of Life (NACEL), 2017 to date	people and those important to them<br>▪ provide audit reports and outputs thereby enabling stakeholders to identify areas for service improvement<br>▪ provide an overview of progress with the provision of high-quality care at the end of life.<br>Timeframe: NCDAH survey of bereaved relatives reviewed care in **last 2 days of life**<br><br>NACEL survey reviewed care **during last admission to hospital**		NACEL surveyed 4,390 bereaved relatives in 2018, with 790 respondents, a response rate of 18% In 2019, 8,783 bereaved relatives invited to take part with 1,581 responding a response rate of 18%	National and Individual results are reported on an interactive online website: The NACEL Quality Survey publish results at national level and an individual hospital level on an interactive online site accessible to healthcare providers participating in the Audit only. All hospitals participating in the NACEL are provided with access to an online benchmarking toolkit with a bespoke dashboard against which they are benchmarked in summary score format. The reporting at individual healthcare provider level allows participating hospitals to utilise evidence from their results to develop actions plans to enhance care at end of life.	people who have died in adult acute hospitals
**Japan:**<br>Japan Hospice and Palliative Care Evaluation (J-HOPE) surveys 2007-2019	The results are used as a source of performance measure for hospice and palliative care services allowing comparison of results from the last places of care focussing on:<br>▪ care satisfaction<br>▪ structure and process of care<br>▪ and achievement of a good death.<br>The J-HOPE4 survey objectives also set out to:<br>▪ examine bereaved family members' self-reported psychosocial conditions, such as grief and depression as bereavement outcomes	**Adult who received hospice and palliative care in all settings** including, designated cancer centres, inpatient palliative care units and home hospice care.	J-Hope surveys of bereaved relatives receiving responses rates of 67% in J-Hope 3 (9,126 completed questionnaires for analysis). In 2015 a response rate of 53%was achieved. In J-Hope 4, 9,071 bereaved relatives responded in 2019.	Individual Reports: Individual reports are provided to each participating institution comparing the quality of care delivered nationally to the care delivered in their own site.<br><br>Individual reports also provide all responses received from bereaved relatives to open ended questions for their service.<br><br>Individual reports are developed by the J-HOPE survey team for the purpose of quality improvement.	Japan Hospice and Palliative Care Evaluation (J-HOPE) surveys are planning the next survey cycle

	▪ provide date for quality improvement purposes for each participating institution<br>▪ provide clinical and academic information concerning the implications of various issues by conducting additional studies.<br>Timeframe: Focussed on delivery of services by **hospice and palliative care services**.				
**Japan:**<br>National mortality follow back survey of bereaved relatives (2018)	The primary objectives:<br>▪ measure the quality of care at end of life for those who died from the leading causes of death in home, hospital and long-term care residential settings<br>▪ test the feasibility of using death registration data to survey about care experience at a national level<br>▪ test the feasibility to assess the quality of care at end of life at a national level<br>▪ examine the respondents' acceptability of this method of survey.<br>Timeframe: Provision of health and social care in **days and weeks before the persons death**	Adult population based survey across **all settings of care**	The survey received 2,294 completed questionnaires for analysis, a response rate of 55.5%	The National Cancer Center Japan published:<br>National Reports and Research Reports in peer reviewed journal publications<br>No Individual reports for quality improvement could not be created for individual hospitals or hospices for a number of reasons including the:<br>▪ difficulty in identifying the different services that the person attended in the days and weeks before the persons death<br>▪ Limits associated with a sample size given the large number of healthcare services, such as small clinics, hospitals, nursing homes etc.	The Medical Support and Partnership division within National Cancer Center Japan are committed with its partners to undertaking further National Mortality follow back surveys on an ongoing basis to ascertain the quality of care delivered from a representative sample of bereaved relatives at a national level

**USA:**<br>Bereaved Family Survey<br>The United States Department of Veterans Affairs (VA)	Survey objectives are:<br>▪ To identify and reduce unwanted variation in the quality of end-of-life care throughout the VA<br>▪ To define and disseminate processes of care ("Best Practices") that contribute to improved outcomes for Veterans near the end of life and their families.<br><br>Timeframe: respondents rate the care provided to the person **in the last month of life**	Adults who died in **all settings of care provided by the VA**	6,000 bereaved relatives responded to the in 2017.<br>Over a four year period in 2009-2013, 43,327 bereaved relatives responded, a 56% response rate.	National Reports and Individual Reports:<br>The aggregated results are reported to each VA medical facility, VA stakeholders, which include VA leadership, policy experts, clinicians, and researchers on a quarterly basis.	The Bereaved Family Survey is undertaken by Veteran Experience Center team on behalf of VA

**USA:**<br>Consumer Assessment of Healthcare Providers and Systems (CAHPS®) Hospice survey	The survey objective is to produce comparable data on the deceased persons' and caregivers' perspectives of care that allow independent, objective and meaningful comparisons across hospice care providers on domains that are important to those receiving the care and create incentives for hospices to improve their quality of care.<br>The survey was developed to:<br>▪ provide a source of information from which selected measures could be publicly reported to beneficiaries and their family members as a decision aid for selection of a hospice program<br>▪ aid hospices with their internal quality improvement efforts and external benchmarking with other facilities<br>▪ provide Centers for Medicare & Medicaid Services (CMS) with information for monitoring the care provided.<br>Timeframe: respondents **rate the hospice and palliative care** provided to the person that died.	**Adults who prior to death received hospice and palliative care in all settings** including, adults hospitals, inpatient palliative care units and home hospice care	Just over 2 million people were surveyed as part of the CAHPS Hospice Survey from April 2017 to March 2019 with a 32.3% response rate	National and Individual results are reported on an interactive online website:<br>Official CAHPS Hospice Survey scores are publicly reported four times each year on the Medicare Care Compare website: www.medicare.gov/care-compare/	The CAHPS Hospice Survey is ongoing. However, due to reporting exemptions granted because of the COVID-19 pandemic, the publicly reported data was frozen with the November 2020 update and will remain frozen until the February 2022 update
