# Supplementary material for: Dying, death and bereavement: developing a national survey of bereaved relatives
Source: BMC Palliat Care. 2023 Feb 23;22:14. doi: 10.1186/s12904-023-01135-2 (PMC9947439; doi:10.1186/s12904-023-01135-2)
Supplement: Supplementary file 3 — Additional file 3: Supplementary information Table 3. Outlines the National End of Life Survey Final Question Bank. [file 12904_2023_1135_MOESM3_ESM.pdf]

Supplementary information table 3:

Supplementary information table 3 outlines the National End of Life Survey Final Question Bank

What is the survey about?

The National End of Life Survey is a nationwide survey that asks about the experiences of care received by you and your relative / friend in the last months and days of life. The results of the survey will be used to improve health and social care services.

We realise that some of the questions may bring back memories that are upsetting, please feel free to skip them and move on to the next question. If the questionnaire raises issues that you would like to talk through, please contact the bereavement support services included with this pack for further support, advice and information.

Why did I get this questionnaire?

You got this questionnaire because you registered the death of a family member or friend in the survey month.

Can I do the questionnaire online?

Yes, please go to **survey.yourexperience.ie** to complete the survey online.

Can I ask someone to help me fill in the survey?

Yes, you may wish to fill the survey with someone else to help you answer some of the questions or you may pass the survey onto the person who knows most about the care provided to the person that died.

Survey Code:

Completing the questionnaire

- As you go through the questionnaire, please follow the instructions and answer the questions by ticking the most appropriate box or boxes like this ☑ using a black or a blue pen.
- Do not worry if you make a mistake; simply fill in the ■ box and put a tick ☑ in the correct box or boxes.
- **There is space at the end of the questionnaire for your written comments.**
- **Please do not write your name or address anywhere on the questionnaire.**
- The questionnaire takes about 30 minutes to complete.
- The term 'healthcare staff' is used to refer to all the staff working in this setting which may include, care workers, healthcare assistants, doctors, nurses, social workers, physiotherapists etc.

Your answers will be treated as strictly confidential. No names will be used in any written reports.

Your feedback is very important and will help us to identify where improvements to health and social care services are necessary.

Thank you for completing the survey.

1. Background information

Q1. What was your relationship to the person that died?

- 1☐ Husband / Civil Partner / Partner
- 2☐ Wife / Civil Partner / Partner
- 3☐ Son / Daughter
- 4☐ Son-in-law / Daughter-in-law
- 5☐ Brother / Sister
- 6☐ Parent
- 7☐ Other relative
- 8☐ Friend / Neighbour
- 9☐ Other:

Q2. This question is about the illnesses the person that died had in the last days and hours of life. Here is a list of illnesses which often affect people towards the end of life.

(Please tick ALL the illnesses she had in the last days of life.)

- 1☐ Cancer (including Leukaemia and Lymphoma)
- 2☐ COVID-19
- 3☐ Dementia
- 4☐ Heart condition (e.g. heart failure)
- 5☐ Influenza or Pneumonia
- 6☐ Lung condition (e.g. COPD)
- 7☐ Neurological condition (including stroke, Motor Neuron Disease)
- 8☐ End stage renal (kidney) disease
- 9☐ I don't know
- 10☐ Something else: ______

Q3. How long had she been ill before she died?

- 1☐ She was not ill — she died suddenly → **GO TO Q107.**
- 2☐ Less than 24 hours
- 3☐ One day or more, but less than one week
- 4☐ One week or more, but less than one month
- 5☐ One month or more, but less than six months
- 6☐ Six months or more but less than 1 year
- 7☐ One year or more

Q4. Did she spend any time being cared for at home during the last three months of life?

- 1☐ Yes → **GO TO Q5.**
- 2☐ No, she was in a hospital, hospice, nursing home or a residential care facility for the whole 3 months → **GO TO Q21.**

2. Care at home

The following questions are about care in the last three months of life at home. Please note that that there are questions later in the questionnaire specifically related to care in the last 2 days of life.

If your relative did not spend any time at home in the last three months of life please go to Q21.

Q5. Did she stay at home during the last three months of her life?

1☐ Yes, she stayed at home.

Please write the name of the town and county where she stayed in the space below:

Town:

County:

2☐ No → **GO TO Q.21**

3☐ Don't know → **GO TO Q.21**

Q6. When she was at home in the last three months of life, did she get care and support at home from any of the services listed below?

These services may be provided by different organisations, such as voluntary organisations, a private agency or the HSE. (Tick all that apply)

1☐ A public health nurse (sometimes called a PHN, district or community nurse)

2☐ A paid carer (sometimes called home help or a care assistant)

3☐ A community palliative care nurse (sometimes called a homecare nurse, or hospice nurse)

4☐ A nurse at night

5☐ A GP (General Practitioner / family doctor)

6☐ Social worker

7☐ Pharmacist

8☐ Occupational therapist (OT)

9☐ Physiotherapist

10☐ She did not receive care and support from these services

11☐ Don't know

Q7. Overall, did you feel she was treated with respect and dignity by the paid carer (sometimes called home help) in the last 3 months of her life?

1☐ Yes, always

2☐ Yes, sometimes

3☐ No

4☐ A paid carer did not visit

5☐ Don't know

Q8. Overall, did you feel she was treated with respect and dignity by the public health nurse in the last 3 months of her life?

1☐ Yes, always
2☐ Yes, sometimes
3☐ No
4☐ A public health nurse did not visit
5☐ Don't know

Q9. Overall, did you feel she was treated with respect and dignity by the GP (family doctor) in the last 3 months of her life?

1☐ Yes, always
2☐ Yes, sometimes
3☐ No
4☐ She did not see the GP
5☐ Don't know

Q10. If she wanted to talk to the GP (family doctor) about her condition, treatment or tests, did she have enough opportunity to do so?

1☐ Yes, definitely
2☐ Yes, to some extent
3☐ No
4☐ She did not want to talk to the GP
5☐ Don't know

Q11. Did someone from a hospice, community palliative care team, or any other specialist palliative care service <u>visit her at home</u> in the last 3 months of her life?

1☐ Yes
2☐ No, this was not needed
3☐ No, this was not offered
4☐ Don't know

Q12. While at home, did healthcare staff help and support her with accessing additional equipment for her home, or making minor adaptations to the home?

1☐ Yes, definitely
2☐ Yes, to some extent
3☐ No
4☐ She did not need additional equipment or adaptations to the home
5☐ Don't know

Q13. While at home, if she experienced pain, did healthcare staff do everything they could to relieve it?

1☐ Yes, definitely
2☐ Yes, to some extent
3☐ No
4☐ She did not have any pain

Q14. While at home, if she experienced symptoms other than pain (such as nausea, constipation, breathing difficulties or restlessness), did healthcare staff do everything they could to relieve them?

1☐ Yes, definitely
2☐ Yes, to some extent
3☐ No
4☐ She did not need help with this

Q15. While at home, did healthcare staff help and support her with her emotional needs (e.g. feeling worried, feeling anxious, feeling low etc.)?

1☐ Yes, definitely
2☐ Yes, to some extent
3☐ No
4☐ She did not need help with this
5☐ Don't know

Q16. While at home, did the healthcare staff help and support her with family worries or concerns (caring for someone else in the family or help with difficult conversations)?

1☐ Yes, definitely
2☐ Yes, to some extent
3☐ No
4☐ She did not need help with this
5☐ Don't know

Q17. While at home, did the healthcare staff give <u>you</u> enough help and support to care for her ?

1☐ Yes, definitely
2☐ Yes, to some extent
3☐ No
4☐ I did not need help with this
5☐ Don't know

Q18. Overall, while she was at home, did the healthcare staff answer your questions in a way that you could understand?

1☐ Yes, always
2☐ Yes, sometimes
3☐ No
4☐ I didn't have any questions

Q19. Overall, while she was at home, did you have confidence and trust in the healthcare staff treating her?

1☐ Yes, always
2☐ Yes, sometimes
3☐ No

Q20. Overall, how would you rate the care she received at home in the last three months?
(Please circle a number)

She had very **poor** care										She had very **good** care
0	1	2	3	4	5	6	7	8	9	10

3. Care in the last nursing home / residential care facility

The following questions are about care provided in a residential care facility, nursing home or long stay care setting during the **last three months of life**. If your relative did not spend any time in one of these settings in the last three months of life please go to Q35.

Please note that that there are questions later in the questionnaire specifically related to care in the last 2 days of life.

Q21 Did she stay in a nursing home / residential care facility during the last three months of her life?

1☐ Yes, she stayed in a nursing home / residential care facility.
Please write the name of the nursing home / residential care facility and the county it is located in the space below:

Name of nursing home/ residential care facility:

☐

County:

☐

2☐ No → **GO TO Q35.**
3☐ Don't know → **GO TO Q35.**

Q22. How long had she stayed in a nursing home / residential care facility before she died?

1☐ Less than 1 week

2☐ 1 to 4 weeks

3☐ 5 to 12 weeks

4☐ If longer than 12 weeks please specify:

Q23. During her stay in the nursing home / residential care facility, if she experienced pain, did healthcare staff do everything they could to relieve it?

1☐ Yes, definitely

2☐ Yes, to some extent

3☐ No

4☐ She did not have any pain

5☐ Don't know

Q24. During her stay in the nursing home / residential care facility, if she experienced symptoms other than pain (such as nausea, constipation, breathing difficulties or restlessness), did healthcare staff do everything they could to relieve them?

1☐ Yes, definitely

2☐ Yes, to some extent

3☐ No

4☐ She did not have any symptoms

5☐ Don't know

Q25. During her stay in the nursing home / residential care facility, did healthcare staff help and support her with her emotional needs (e.g. feeling worried, feeling anxious, feeling low etc.)?

1☐ Yes, definitely

2☐ Yes, to some extent

3☐ No

4☐ She did not need help with this

5☐ Don't know

Q26. During her stay in the nursing home / residential care facility, did healthcare staff help her with family worries or concerns (caring for someone else in the family or help with difficult conversations)?

1☐ Yes, definitely

2☐ Yes, to some extent

3☐ No

4☐ She did not need help with this

5☐ Don't know

Q27. During her stay in the nursing home / residential care facility, did healthcare staff support her to do things she wanted to do (for example, participate in special family events, seeing a pet etc.)?

1☐ Yes, definitely

2☐ Yes, to some extent

3☐ No

4☐ She did not need support with this

5☐ Don't know

Q28. During her stay in the nursing home / residential care facility, did healthcare staff provide help to her with urgent problems outside of normal office hours (during the evenings after 5pm, at night and weekends)?

1☐ Yes, definitely
2☐ Yes, to some extent
3☐ No
4☐ She did not need help with this
5☐ Don't know

Q29. During her stay in the nursing home / residential care facility, if she needed help from the healthcare staff, did she get it as soon as she needed it?

1☐ Yes, always
2☐ Yes, sometimes
3☐ No
4☐ She did not need help
5☐ Don't know

Q30. During her stay in the nursing home / residential care facility, did healthcare staff answer your questions in a way that you could understand?

1☐ Yes, completely
2☐ Yes, to some extent
3☐ No
4☐ I didn't have any questions

Q31. During her stay in the nursing home / residential care facility were you made to feel welcome to visit at any time?

1☐ Yes, always
2☐ Yes, sometimes
3☐ No
4☐ I wasn't allowed due to visiting restrictions

Q32. During her stay in the nursing home / residential care facility, did you have confidence and trust in the healthcare staff treating her?

1☐ Yes, always
2☐ Yes, sometimes
3☐ No

Q33. Overall, during her stay in the nursing home / residential care facility, did you feel she was treated with respect and dignity by the healthcare staff?

1☐ Yes, always
2☐ Yes, sometimes
3☐ No
4☐ Don't know

Q34. Overall, how would you rate the care she recieved in the nursing home / residential care facility?

(Please circle a number)

She had very **poor** care — She had very **good** care

0	1	2	3	4	5	6	7	8	9	10

4. Care during the last hospital stay

The following questions are about care in an acute hospital (this is a hospital that has an Emergency Department or A&E or Urgent Care Centre). If your relative did not spend any time in an acute hospital in the last three months of life please go to Q. 49.

The questions that follow in this section are about care during the last hospital stay. Please note that that there are questions later in the questionnaire specifically related to care in the last 2 days of life.

Q35. Did she stay in an acute hospital at any time during her last three months of life?

1☐ Yes, she stayed in an acute hospital.
Please write the **name of the last acute hospital** she stayed in the space below:

Name of hospital:

County:

2☐ No → **GO TO Q49.**

3☐ Don't know → **GO TO Q49.**

Q36. How many times had she been in hospital in the last three months including the last admission to hospital?

1☐ Once

2☐ Twice

3☐ 3 or more times

4☐ Not sure

Q37. How long had she stayed in the hospital during her last admission?

1☐ Less than 1 week

2☐ 1 to 4 weeks

3☐ 5 to 12 weeks

4☐ If longer than 12 weeks please specify:

Q38. During this last admission to hospital, if she experienced pain, did healthcare staff do everything they could to relieve it?

1☐ Yes, definitely

2☐ Yes, to some extent

3☐ No

4☐ She did not have any pain

5☐ Don't know

Q39. During this last admission to hospital, if she experienced symptoms other than pain (such as nausea, constipation, breathing difficulties or restlessness), did healthcare staff do everything they could to relieve them?

1☐ Yes, definitely

2☐ Yes, to some extent

3☐ No

4☐ She did not have any symptoms

5☐ Don't know

Q40. During this last admission to hospital did healthcare staff help and support her with her emotional needs (e.g. feeling worried, feeling anxious, feeling low etc.)?

1☐ Yes, definitely

2☐ Yes, to some extent

3☐ No

4☐ She did not need help with this

5☐ Don't know

Q41. During this last admission to hospital, did healthcare staff help and support her with family worries or concerns (caring for someone else in the family or help with difficult conversations)?

1☐ Yes, definitely

2☐ Yes, to some extent

3☐ No

4☐ She did not need help with this

5☐ Don't know

Q42. During this last admission to hospital, did healthcare staff provide enough help with urgent problems outside of normal office hours (during the evenings after 5pm, at night and weekends)?

1☐ Yes, definitely

2☐ Yes, to some extent

3☐ No

4☐ She did not need help with this

5☐ Don't know

Q43. During this last admission to hospital, if she needed help from the healthcare staff, did she get it as soon as she needed it?

1☐ Yes, always

2☐ Yes, sometimes

3☐ No

4☐ She did not need help

5☐ Don't know

Q44. During this last admission to hospital, did healthcare staff answer your questions in a way that you could understand?

1☐ Yes, completely

2☐ Yes, to some extent

3☐ No

4☐ I didn't have any questions

Q45. During this last admission to hospital, were you made to feel welcome to visit at any time?

1☐ Yes, always

2☐ Yes, sometimes

3☐ No

4☐ Not applicable

5☐ I wasn't allowed due to visiting restrictions

Q46. During this last admission to hospital, did you have confidence and trust in the healthcare staff treating her?

1☐ Yes, always

2☐ Yes, sometimes

3☐ No

Q47. Overall, did you feel she was treated with respect and dignity by the hospital staff during this last admission to hospital?

1☐ Yes, always

2☐ Yes, sometimes

3☐ No

4☐ Don't know

Q48. Overall, how would you rate the care she recieved in the hospital?

(Please circle a number)

She had very **poor** care — She had very **good** care

0	1	2	3	4	5	6	7	8	9	10

5. Care during the last hospice stay

The following questions are about the care provided to people who stayed in a hospice in the last three months. If your relative did not spend any time staying overnight in a hospice in the last three months of life please go to Q.63

The questions are about care during the last hospice stay. Please note that that there are questions later in the questionnaire specifically related to care in the last 2 days of life.

Q49. Did she stay in a hospice at any time during her last three months of life?

1☐ Yes

If 'Yes' please write the name and location of the last hospice she stayed in the space below:

Name of hospice:

☐

County:

☐

3☐ No → **GO TO Q63.**

4☐ Don't know → **GO TO Q63.**

Q50. How long had she stayed in the hospice during her last admission?

1☐ Less than 1 week

2☐ 1 to 4 weeks

3☐ 5 to 12 weeks

4☐ If longer than 12 weeks please specify:

☐

Q51. **During her stay in the hospice, if she experienced pain, did healthcare staff do everything they could to relieve it?**

1☐ Yes, definitely
2☐ Yes, to some extent
3☐ No
4☐ She did not have any pain
5☐ Don't know

Q52. **During her stay in the hospice, if she experienced symptoms other than pain (such as nausea, constipation, breathing difficulties or restlessness), did healthcare staff do everything they could to relieve them?**

1☐ Yes, definitely
2☐ Yes, to some extent
3☐ No
4☐ She did not have any symptoms
5☐ Don't know

Q53. **During her stay in the hospice, did healthcare staff help and support her with her emotional needs (e.g. feeling worried, feeling anxious, feeling low etc.)?**

1☐ Yes, definitely
2☐ Yes, to some extent
3☐ No
4☐ She did not need help with this
5☐ Don't know

Q54. **During her stay in the hospice, did healthcare staff help and support her with family worries or concerns (caring for someone else in the family or help with difficult conversations)?**

1☐ Yes, definitely
2☐ Yes, to some extent
3☐ No
4☐ She did not need help with this
5☐ Don't know

Q55. **During her stay in the hospice, did healthcare staff support her to do things she wanted to do, (for example, participate in special family events, seeing a pet etc.)?**

1☐ Yes, definitely
2☐ Yes, to some extent
3☐ No
4☐ She did not need support with this
5☐ Don't know

Q56. **During her stay in the hospice, did healthcare staff provide help with urgent problems outside of normal office hours (during the evenings after 5pm, at night and weekends)?**

1☐ Yes, definitely
2☐ Yes, to some extent
3☐ No
4☐ She did not need help with this
5☐ Don't know

Q57. **During her stay in the hospice, if she needed help from the healthcare staff, did she get it as soon as she needed it?**

1☐ Yes, always
2☐ Yes, sometimes
3☐ No
4☐ She did not need help
5☐ Don't know

Q58. **During her stay in the hospice, were you made to feel welcome to visit at any time?**

1☐ Yes, always
2☐ Yes, sometimes
3☐ No
4☐ I wasn't allowed due to visiting restrictions

Q59. **During her stay in the hospice, did healthcare staff answer your questions in a way that you could understand?**

1☐ Yes, completely

2☐ Yes, to some extent

3☐ No

4☐ I didn't have any questions

Q60. **During her stay in the hospice, did you have confidence and trust in the healthcare staff treating her?**

1☐ Yes, always

2☐ Yes, sometimes

3☐ No

Q61. **Overall, during her stay in the hospice, did you feel she was treated with respect and dignity by the healthcare staff?**

1☐ Yes, always

2☐ Yes, sometimes

3☐ No

4☐ Don't know

Q62. **Overall, how would you rate the care she recieved in the hospice?**

(Please circle a number)

She had very **poor** care										She had very **good** care
0	1	2	3	4	5	6	7	8	9	10

6. Coordination of care during the last 3 months of life

The following questions are about the coordination of care during the last three months of life.

Q63. Did you feel that there was good coordination between different services (e.g. GP, hospital, nursing home or hospice) and healthcare staff that cared for her in the last three months of her life?

1☐ Yes, definitely

2☐ Yes, to some extent

3☐ No

Q64. Is there anything you would like to tell us about her experiences of transferring between different settings (for example from home to hospital, etc.)?

____
____
____
____
____
____
____
____
____
____
____
____
____
____
____
____

7. Care experiences in the last two days of life

The following questions are about the care experience in the last two days of life. The next sections are a sensitive part of the questionnaire and it can be emotional considering care at this time. We appreciate you taking the time to answer the questions, as the answers will help us understand the experiences of care at this significant time.

If you had no contact with her in the last two days of life, please go to Q. 95

Q65. During the last two days of her life, was she cared for:

1☐ At home or in the home of a family member or friend.

2☐ In a nursing home / residential care facility

3☐ In an acute hospital (a hospital that has an Emergency Department or A&E or Urgent Care Centre)

4☐ In a hospice

5☐ Other – please feel free to comment below:

Q66. During the last two days of her life, if she experienced pain, did healthcare staff do everything they could to relieve it?

1☐ Yes, definitely

2☐ Yes, to some extent

3☐ No

4☐ She did not have any pain

5☐ I was not with her in the last two days → **PLEASE GO TO Q95.**

Q67. During the last two days of her life, if she experienced symptoms other than pain (such as nausea, constipation, breathing difficulties or restlessness), did healthcare staff do everything they could to relieve them?

1☐ Yes, definitely

2☐ Yes, to some extent

3☐ No

4☐ She did not have any symptoms

Q68. During the last two days of her life, was there enough help available to meet her personal care needs (such as washing, dressing and toileting)?

1☐ Yes, definitely

2☐ Yes, to some extent

3☐ No

4☐ She did not need help with personal care needs

Q69. During the last two days of her life, was there was enough help with nursing care, such as giving medicine and helping her find a comfortable position in bed?

1☐ Yes, definitely

2☐ Yes, to some extent

3☐ No

4☐ Don't know

Q70. During the last two days of her life, did healthcare staff help and support her with her emotional needs (e.g. feeling worried, feeling anxious, feeling low etc.)?

1☐ Yes, definitely

2☐ Yes, to some extent

3☐ No

4☐ She did not need help with this

5☐ Don't know

Q71. During the last two days of her life, did healthcare staff provide support to meet her religious or spiritual needs (things like talking, praying, quiet time or access to a religious or spiritual leader)

1☐ Yes, always
2☐ Yes, sometimes
3☐ No
4☐ She did not need help with this
5☐ Don't know

Q72. During the last two days of her life, did she get enough help from healthcare staff to eat or drink if she wished?

1☐ Yes, always
2☐ Yes, sometimes
3☐ No
4☐ She did not want help
5☐ She was not able to eat or drink
6☐ Don't know

Q73. During the last two days of her life, did the healthcare staff take account of her individual requirements when planning her care?

1☐ Yes, definitely
2☐ Yes, to some extent
3☐ No
4☐ Don't know

Q74. During the last two days of her life, did someone from the hospice, palliative care team, or any other specialist palliative care service visit her?

1☐ Yes
2☐ No
3☐ Don't know

Q75. During the last two days of her life, did you feel that there was good coordination between different members of healthcare staff?

1☐ Yes, definitely
2☐ Yes, to some extent
3☐ No
4☐ Don't know

Q76. During the last two days of her life, was there adequate support to meet her needs outside of outside of normal office hours (during the evenings after 5pm, at night and weekends)?

1☐ Yes, always
2☐ Yes, sometimes
3☐ No
4☐ Don't Know

Q77. During the last two days of her life, do you think she was involved as much as she wanted to be in decisions about her care and treatment?

1☐ Yes, definitely
2☐ Yes, to some extent
3☐ No
4☐ She was not able to be involved in decisions at this time
5☐ Don't know

Q78.

During the last two days of her life, did healthcare staff discuss with <u>you</u> the level of treatment and care and its appropriateness (in terms of medical interventions and their illness) that your relative or friend would receive?

1☐ Yes, definitely
2☐ Yes, to some extent
3☐ No
4☐ Don’t Know

Q79. During the last two days of her life, if she agreed, did healthcare staff keep <u>you</u> informed on her condition and care?

1☐ Yes, definitely
2☐ Yes, to some extent
3☐ No
4☐ She did not agree to the healthcare staff talking to me about her condition and care

---

Q80. During the last two days of her life, did healthcare staff explain her condition and care in a way that <u>you</u> could understand?

1☐ Yes, definitely
2☐ Yes, to some extent
3☐ No

Q81. During the last two days of her life, did <u>you</u> feel you had enough time to discuss her care and treatment with healthcare staff?

1☐ Yes, definitely
2☐ Yes, to some extent
3☐ No
4☐ Don’t Know

Q82. During the last two days of her life, did you have confidence and trust in the *<u>nurses</u>* who were caring for her?

1☐ Yes, always
2☐ Yes, sometimes
3☐ No
4☐ Don’t Know

Q83. During the last two days of her life, did you have confidence and trust in the *<u>doctors</u>* who were caring for her?

1☐ Yes, always
2☐ Yes, sometimes
3☐ No
4☐ Don’t Know

Q84. During the last two days of life, do you think the care provided by the healthcare staff met her individual needs?

1☐ Yes, definitely
2☐ Yes, to some extent
3☐ No

Q85. During the last two days of her life, did the healthcare staff treat her with kindness and compassion?

1☐ Yes, always
2☐ Yes, sometimes
3☐ No

Q86. Overall, during the last two days of life, did you feel that she was treated with respect and dignity by the healthcare staff?

1☐ Yes, always
2☐ Yes, sometimes
3☐ No
4☐ Don’t know

Q87. **Is there anything else you would like to tell us about the care she received in the last two days of her life? Please feel free to comment below:**

8. Care in the last two days of life in a healthcare facility

The following questions are about care in a healthcare facility such as a hospital, hospice, nursing home, residential care facility or long stay care setting in the last two days of life.

If she did not spend any time in one of these settings in the last two days of life or if you had no contact with her in the last two days of life, please go to Q. 95.

Q88. What type of room was she cared for in when she died ?

1☐ Single room → **PLEASE GO TO Q90.**

2☐ Ward or room shared with other people

3☐ Other

4☐ She died at home → **PLEASE GO TO Q95.**

Q89. Was she offered the choice of moving to a single room?

1☐ Yes

2☐ No, that choice was not offered or available

3☐ No, she was already in a single room

4☐ Don't know

Q90. In the last two days of her life, did the bed area and surrounding environment have adequate privacy for her?

1☐ Yes, definitely

2☐ Yes, to some extent

3☐ No

Q91. In the last two days of her life, was there a designated family room for you to meet with healthcare staff with adequate peace and privacy?

1☐ Yes, definitely

2☐ Yes, to some extent

3☐ No

Q92. During the last two days of her life, were you told you could visit at any time including outside of regular visiting hours?

1☐ Yes

2☐ No

Q93. During the last two days of her life, were you given the option to stay overnight in the healthcare facility (hospital, hospice, nursing home) at any time?

1☐ Yes

2☐ No

3☐ Don't Know

Q94. During the last two days of her life, did you have access to a family room with tea and coffee making facilities?

1☐ Yes

2☐ No

3☐ Don't Know

9. Your experience of care and support

The following questions are about your experience of the care and support provided to you by the healthcare staff.

Q95. Looking back over the last days of her life, were you given enough emotional help and support (e.g. feeling worried, feeling anxious, feeling low etc.) by the healthcare staff ?

1☐ Yes, definitely
2☐ Yes, to some extent
3☐ No
4☐ I did not need help with this
5☐ Not applicable, I didn't have any contact with healthcare staff

Q96. Looking back over the last days of her life, were you given enough help and support by the healthcare staff to talk to children or young adults about her illness?

1☐ Yes, definitely
2☐ Yes, to some extent
3☐ No
4☐ I did not need help with this
5☐ Not applicable, I didn't have any contact with healthcare staff

Q97. Looking back over the last days of her life, did the healthcare staff listen carefully to you?

1☐ Yes, definitely
2☐ Yes, to some extent
3☐ No
4☐ Not applicable, I didn't have any contact with healthcare staff

Q98. Looking back over the last days of her life, were you involved as much as you wanted to be in decisions about her care and treatment?

1☐ Yes, definitely
2☐ Yes, to some extent
3☐ No
4☐ Not applicable, I didn't have any contact with healthcare staff

Q99. Overall, how would you rate the care and support you received? (Please circle a number)

I got very **poor** care and support										I got very **good** care and support
0	1	2	3	4	5	6	7	8	9	10

10. Circumstances surrounding her death

Q100. Was she told by a healthcare professional that she was likely to die?

1☐ Yes
2☐ No →**GO TO Q102.**
3☐ Don't know →**GO TO Q102.**

Q101. In your opinion, did the healthcare professional who told her that she was likely to die break the news to her in a sensitive and caring way?

1☐ Yes, definitely
2☐ Yes, to some extent
3☐ No
4☐ Don't know

Q102. Did the healthcare professional who told you she was likely to die break the news to you in a sensitive and caring way?

1☐ Yes, definitely
2☐ Yes, to some extent
3☐ No
4☐ I was not told she was likely to die

Q103. Did the healthcare staff answer your questions in a way that you could understand?

1☐ Yes, definitely
2☐ Yes, to some extent
3☐ No
4☐ I didn't have any questions

Q104. Did the healthcare staff talk to you about what to expect when she was dying (for example symptoms that may arise)?

1☐ Yes, definitely →**GO TO Q106.**
2☐ Yes, to some extent →**GO TO Q106.**
3☐ No →**GO TO Q105.**
4☐ Not applicable – she died unexpectedly →**GO TO Q106.**

Q105. Would a discussion about what to expect when she was dying have been helpful?

1☐ Yes
2☐ No

Q106. (a) Were you contacted soon enough to give you time to be with her before she died?

1☐ Yes
2☐ No

Q106. (b) If 'No', which of the following applied?

1☐ It was not clear that she was going to die soon
2☐ I wasn't informed that she was going to die
3☐ I should have been contacted earlier by staff
4☐ I couldn't have got there anyway
5☐ I wasn't allowed to be there due to visiting restrictions
6☐ Other reason

Q107. Where did she die?

1☐ In her own home or in the home of a family member or friend

Name of Town and County:

2☐ In a hospital – please write the name of the hospital below:
Name of hospital:
County:

3☐ In a hospice – please write the name of the hospice below
Name of hospice:
County:

4☐ In a nursing home / residential care facility – please write the name of the facility below:
Name:
County:

5☐ In an ambulance on the way to hospital / hospice

6☐ Somewhere else (please write below):

Q108. Were you given enough help and support by the healthcare staff at the actual time of her death?

1☐ Yes, definitely

2☐ Yes, to some extent

3☐ No

4☐ Not applicable

Q109. Did the healthcare staff give you practical information about what to do after she died, including information on registering the death?

1☐ Yes, definitely

2☐ Yes, to some extent

3☐ No

4☐ I did not need this information

5☐ Not applicable, I didn't have any contact with the healthcare staff

Q110. After she died, did the healthcare staff deal with you in a sensitive manner?

1☐ Yes, definitely

2☐ Yes, to some extent

3☐ No

4☐ Not applicable, I didn't have any contact with the healthcare staff

Q111.

(a) Since she died, have you talked to anyone from health and social work services, or from a bereavement service, about your feelings about her illness and death?

1☐ Yes

2☐ No, but I would have liked to

3☐ No, but I did not want to talk to anyone about this

Q111. If 'yes' to whom did you speak to?

(b) (Tick all that apply)

1☐ GP / Doctor

2☐ Nurse

3☐ Social worker

4☐ Pastoral care or healthcare chaplain

5☐ Bereavement Counsellor

6☐ Bereavement helpline

7☐ Not sure

8☐ Other

Q112. On balance, do you think that she died in the right place?

1☐ Yes

2☐ No

3☐ Not sure

Q113. Overall, how would you rate the care your relative received at the end of life? (Please circle a number)

She had very **poor** care										She had very **good** care
0	1	2	3	4	5	6	7	8	9	10

11. Other Comments

Thank you very much for taking part in this survey. We would like to give you the opportunity to tell us in your own words about the care you and the person that died received. To do this, you may like to answer the questions below. You can use the back page of the questionnaire if you need more space. Comments will be entered into a secure database after removing any information that could identify you.

This anonymised feedback will be looked at by HIQA, the HSE and the Department of Health to try to understand and improve experiences of health and social care at end of life. Other researchers may also analyse anonymised data from this survey in the future, after all personal information that could identify you has been removed. We will give examples of feedback in the final survey reports to provide a fuller understanding of bereaved relatives' experiences.

Q114 **Overall, what was good about her care?**

Q115 **Was there anything that could be improved?**

Q116 **Do you have any other comments or suggestions about the care she received?**

12. Information about you both

In this section, we would like to know a little more about you both. This will help us make better use of the information you have given us.

Q117. The main person who filled in this questionnaire is :

1☐ Male

2☐ Female

3☐ Other

4☐ Prefer not to say

Q118. How old are you?

1☐ 18-29 years

2☐ 30-39 years

3☐ 40-49 years

4☐ 50-59 years

5☐ 60-69 years

6☐ 70-79 years

7☐ 80-89 years

8☐ 90 + years

Q119. What is your ethnic or cultural background?
(Tick **ONE** box only)

White:

1☐ Irish

2☐ Irish Traveller

3☐ Roma

4☐ Any other White background

Black or Black Irish:

5☐ African

6☐ Any other Black background

Asian or Asian Irish:

7☐ Chinese

8☐ Indian/ Pakistani/ Bangladeshi

9☐ Any other Asian background

Other, including mixed group/ background:

10☐ Arabic

11☐ Mixed, *please specify:*

12☐ Other, *please write your ethnic group here:*

Q120. The person that died was:

1☐ Male

2☐ Female

3☐ Other

4☐ Prefer not to say

Q121. Please indicate the ethnic background of the person that died:

(Tick **ONE** box only)

White:

1☐ Irish

2☐ Irish Traveller

3☐ Roma

4☐ Any other White background

Black or Black Irish:

5☐ African

6☐ Any other Black background

Asian or Asian Irish:

7☐ Chinese

8☐ Indian/ Pakistani/ Bangladeshi

9☐ Any other Asian background

Other, including mixed group/ background:

10☐ Arabic

11☐ Mixed, *please specify:* ________

12☐ Other, *please write your ethnic group here:* ________

Q122. What was her age when she died?

(Please enter their age in numbers)

________ Years old

Q123. Did the person that died have :

1☐ A medical card?

2☐ Private health insurance?

3☐ **Both** a medical card and private health insurance?

4☐ **Neither** a medical card nor private health insurance?

If you would like to talk about your feelings or discuss upsetting memories brought back by completing this questionnaire, please feel free to contact the Irish Hospice Foundation Bereavement Support freephone service on 1800 80 70 77 to talk to a bereavement support volunteer.

THANK YOU VERY MUCH FOR COMPLETING THE SURVEY

Please check that you have answered all of the questions that apply to you.

Please return this questionnaire in the Freepost envelope provided. No stamp is needed.
